# PReS-FINAL-2294: Juvenile systemic lupus erythematosus in ethnically homogeneous population: a single-centre experience

**DOI:** 10.1186/1546-0096-11-S2-P284

**Published:** 2013-12-05

**Authors:** H Malcova, D Nemcova, J Hoza, P Dolezalova

**Affiliations:** 1 Department of Paediatrics, University Hospital Motol, Prague, Czech Republic; 2 Paediatric Rheumatology Unit, Department of Paediatrics and Adolescent Medicine, General University Hospital, Prague, Czech Republic

## Introduction

Due to its clinical heterogeneity and unpredictable course childhood systemic lupus erythematosus (JSLE) represents one of the most challenging conditions in rheumatology. We have audited our unit's clinical practice in order to determine not only characteristics of our patient cohort, but also to identify potential for our clinical care improvement.

## Objectives

To evaluate clinical presentation, therapeutic approaches and disease outcome in a cohort of children with JSLE in a single central European centre.

## Methods

Electronic records of patients attending the Unit over the period from January 2005 (when hospital information system was established) to April 2012 were reviewed. Inclusion criteria were diagnosis of SLE according to the modified ACR classification criteria in patients aged ≤18 years and active disease requiring daily steroid dose higher than 5 mg. Data on clinical and laboratory findings and treatment modalities from the entry and the last clinic visits were analyzed.

## Results

27 cases were identified, 26 Caucasians, 23 (85%) females, with the mean age at the diagnosis 14,1 years (± 3,3). In 22 cases JSLE diagnosis was made within the analyzed time period, in 5 patients with earlier disease onset older data were retrieved from the notes. Mothers of 2 patients suffered with SLE as well. Patients were followed for a mean period of 3,7 years (± 2,2). At the diagnosis, 17 (63%) patients had skin involvement, 18 (67%) musculoskeletal manifestations and 4 (15%) neurological involvement. Patients presented with cytopenia (n = 16; 59%), positive ANA (n = 25;93%) and anti-dsDNA (n = 18;67%). In 10 patients (37%) renal involvement was noted, in 4 patients at disease onset, in 2 cases during the first year of the disease and in 4 cases nephritis evolved after the mean of 3,8 years from onset. Laboratory characteristics of antiphospholipid syndrome were present in 17 (63%) patients, 5 had thromboembolic complications and 3 had chorea. All patients were treated with corticosteroids, most of them in combination with hydroxychloroquine (93%), azathioprine and methotrexate were administered to 11 (41%) and 7 (26%) patients respectively, 4 patients were treated with cyclosporine, 6 with i.v. cyclophosphamide and 1 with mycophenolate mofetil. At the time of data analysis 13 patients were still followed by us, 4 of them were in clinical remission on maintenance therapy and 9 had mild or moderate disease activity on combined immunosuppression. Transition to rheumatology service was completed in 14 patients at the mean age of 19 years, 10 of them had still active disease at the time of transfer.

## Conclusion

Number of patients followed at our Unit reflects low incidence of SLE in our population (total 10 million) and underlines importance of centralised care in order to accumulate and maintain relevant disease management expertise. Having analysed our records critically we have noted paucity of important data including patient-reported disease outcomes as well as systematic disease assessments (SLEDAI, SLICC). Despite this we have been able to demonstrate distribution of organ involvement and disease severity as reflected in treatments used. Although none of the patients died during the follow-up, 19/27 had ongoing active disease at the final assessment.

## Disclosure of interest

None declared.

